# Characterization of a new commercial single crystal diamond detector for photon- and proton-beam dosimetry

**DOI:** 10.1093/jrr/rrv044

**Published:** 2015-08-12

**Authors:** Yuichi Akino, Archana Gautam, Len Coutinho, Jan Würfel, Indra J. Das

**Affiliations:** 1 Department of Radiation Oncology, Indiana University School of Medicine, Indianapolis 46202, USA; 2 Department of Radiation Oncology, Indiana University Health Proton Therapy Center, Bloomington 47408, USA; 3 PTW-Freiburg GmbH, Loerracher Strasse 7, Freiburg 79115, Germany; 4 Present address: Division of Health Sciences, Osaka University Graduate School of Medicine, 1–7 Yamadaoka, Suita, Osaka 565–0871, Japan; 5 Present address; Department of Radiation Physics, MD Anderson Cancer Center, 1515 Holocombe Blvd, Houston, Tx 77030, USA

**Keywords:** detector, microdiamond, dosimetry, characterization

## Abstract

A synthetic single crystal diamond detector (SCDD) is commercially available and is characterized for radiation dosimetry in various radiation beams in this study. The characteristics of the commercial SCDD model 60019 (PTW) with 6- and 15-MV photon beams, and 208-MeV proton beams, were investigated and compared with the pre-characterized detectors: Semiflex (model 31010) and PinPoint (model 31006) ionization chambers (PTW), the EDGE diode detector (Sun Nuclear Corp) and the SFD Stereotactic Dosimetry Diode Detector (IBA). To evaluate the effects of the pre-irradiation, the diamond detector, which had not been irradiated on the day, was set up in the water tank, and the response to 100 MU was measured every 20 s. The depth–dose and profiles data were collected for various field sizes and depths. For all radiation types and field sizes, the depth–dose data of the diamond chamber showed identical curves to those of the ionization chambers. The profile of the diamond detector was very similar to those of the EDGE and SFD detectors, although the Semiflex and PinPoint chambers showed volume-averaging effects in the penumbrae region. The temperature dependency was within 0.7% in the range of 4–41°C. A dose of 900 cGy and 1200 cGy was needed to stabilize the chamber to the level within 0.5% and 0.2%, respectively. The PTW type 60019 SCDD detector showed suitable characteristics for radiation dosimetry, for relative dose, depth–dose and profile measurements for a wide range of field sizes. However, at least 1000 cGy of pre-irradiation will be needed for accurate measurements.

## INTRODUCTION

Finding a perfect detector with wide applications in radiation fields has always been elusive. Improvements in detector characteristics for radiation (photon, electron and protons), dose, dose rate and fields (small and large) has been steadily pursued. Advances in treatment techniques like stereotactic radiotherapy (SRT), CyberKnife, Gamma Knife, Tomotherapy, intensity-modulated radiotherapy (IMRT) and volumetric-modulated arc therapy (VMAT) have created an urgency for suitable detectors for small-field dosimetry [1–3]. Accurate dosimetry of small fields is challenging because the finite range of detectors leads to volume-averaging effects with various types of perturbations [4–8]. A number of detectors (including small ionization chambers, diode detectors, and diamond detectors) have been investigated with limited success or limited scope [9, 10].

Natural diamond detectors have been extensively studied and found to have suitable characteristics for dosimetry [11–16]. Unfortunately, natural diamond detectors, even those with superior characteristics, have become obsolete due to poor design, selection of crystal, and craftsmanship and to cost. Although synthetic diamonds produced by chemical vapor deposition (CVD) [17, 18] have been considered for small-field dosimetry, some problems, including the difficulties in controlling the incorporation of impurities into synthetic crystals, and encapsulation have limited the performance of the detector. Recently, a new single crystal diamond detector (SCDD) was developed at the laboratories of the University of Rome ‘Tor Vergata’. Ciancaglioni *et al.* reported the characteristics of the SCDD for small-field dosimetry [19]. Several studies investigating the dosimetry of photon, electron and proton beams have also been reported [9, 10, 20, 21]. The prototype of the SCDD was embedded in a polymethyl-methacrylate (PMMA) waterproof cylindrical housing [19–22] or in the same PTW housing used for the unshielded Silicon diode model 60017 (PTW-Freiburg GmbH, Freiburg, Germany) [23–25]. Currently, a commercial product model of the SCDD (Model 60019, PTW) is available. Several studies have investigated the characteristics of the commercial model for electron beams [24, 26] and small-field photon dosimetry [27, 28]. Although the characteristics of the prototype SCDD for proton dosimetry are reported by Mandapaka *et al.* [20], those of the commercial model have not been reported. In addition, some characteristics for photon dosimetry (such as pre-irradiation) showed variation among previous reports. In this study, we have reported the characteristics of the commercial SCDD model 60019 for uniform-scanning proton-beam dosimetry and also carried out a re-investigation of megavoltage photon dosimetry.

## MATERIALS AND METHODS

### Diamond detector

**Fig. 1.**
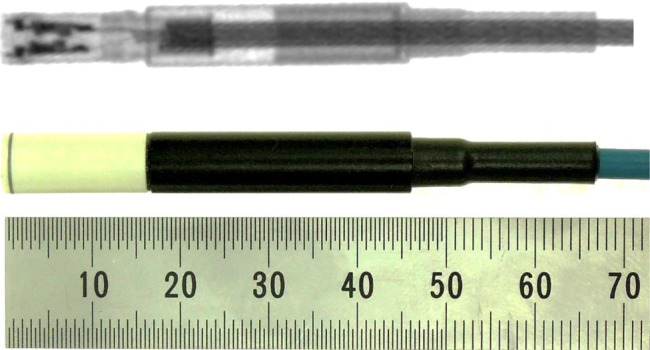
Photo and X-ray image of the PTW Type 60019 detector.

A photo and radiographic images of the microDiamond model 60019 (PTW) are shown in Fig. 1. The detector's sensitive volume is 0.004 mm^3^ and is of a circular shape with a 1.1-mm radius and 1-μm thickness. In all of the measurements in this study, the SCDD was operated in photovoltaic mode, i.e. with no external bias voltage applied. The shape of SCDD is cylindrical, 7 mm in diameter and 45.5 mm in length. The water-equivalent entrance window (1.0-mm thickness) consists of 0.3 mm of RW3, 0.6 mm of Epoxy, and 0.01 mm of Aluminum. A thorough study of the physical properties and detection mechanism of such a device is reported elsewhere [11].

### Measurements

A uniform-scanning proton-beam (USPB) was investigated in this study. The initial energy of the proton beam was 208 MeV. A detailed description of the USPB has been provided elsewhere [29, 30]. The characteristics of the SCDD with a USPB beam was compared with that of a plane-parallel PTW 34045 Markus ionization chamber. The SCDD and Markus chamber were mounted on the arm of a Blue Phantom scanning water phantom (IBA Dosimetry, GmbH, Schwarzenbruck, Germany). The proton beam was irradiated from 270° direction (IEC convention). The air gap was 5 cm. For percentage depth dose (PDD) measurements, 100 MU of pristine Bragg peaks with the energy range of 8, 16 and 24 cm was measured at various depths because a continuous scanning measurement is not suitable for USPB. To investigate the beam linearity of the response, proton beams with various monitor units (10–1000 MU) were measured at the center of the spread-out Bragg peak (SOBP). Dose-rate dependency was also evaluated by measurements of the proton beam with various beam current (equivalent to 0.5–6 Gy/min dose rate).

The characteristics of the SCDD for photon beam dosimetry were also investigated for 6- and 15-MV photon beams generated by a Clinac 2100C/D linear accelerator (Varian Medical Systems, Palo Alto, USA). An MP3 water phantom scanning system (PTW) was used for measurements, with its surface at a source–surface distance = 100 cm. The dose linearity of the response of each detector was evaluated by measuring 3–1000 MU with a 10 × 10 cm^2^ field size at 10 cm depth. The measurements were performed for the dose rates of 100, 300 and 500 MU/min in order to evaluate the dose-rate dependency. The PDD and off-center ratio (OCR) data were collected for 3 × 3 cm^2^, 10 × 10 cm^2^, 20 × 20 cm^2^ and 30 × 30 cm^2^ field sizes. Profile data were analyzed using an Akilles in-house software.

To compare the characteristics of SCDD with those of other pre-characterized detectors, Semiflex (model 31010, sensitive volume of 0.125 cm^3^) and PinPoint (model 31006, sensitive volume of 0.016 cm^3^) ionization chambers (PTW), the EDGE diode detector (Sun Nuclear Corp, Melbourne, USA) and the SFD Stereotactic Dosimetry Diode Detector (IBA Dosimetry) were also used for measurements with the same settings. The Semiflex ionization chamber was considered to provide the reference data in this study because of its typical sensitive volume for data collection for the commissioning. A CNMC model K602 electrometer (CNMC Co., Nashville, U.S.A.) was used for point dose measurement. For the ionization chambers, measurements were conducted under an applied +300 V. For the other detectors (SCDD, EDGE, and SFD), no bias was applied.

The temperature dependency was tested in water for the range of 4–60°C. The diamond detector was hung vertically with a metallic stand, and the sensitive volume of the chamber was positioned at the isocenter of the linac in a plastic case filled with ice-cold water. The metallic holder was at least 20 cm away from the sensitive volume of SCDD to eliminate any effects due to scattered radiation from the metallic stand. The response of the chamber to 100 MU of a 6-MV photon beam was evaluated for various water temperatures by replacing a part of the water with hot water, keeping the volume of water in the plastic case constant. After stirring the water, the water temperature was measured at several positions using an electric thermometer. To evaluate the effects of the pre-irradiation, the diamond detector, which had not been irradiated on the day, was set up in the water tank and the response to 100 MU was measured every 20 s. The measurements were repeated on three different days. After enough irradiation for stabilizing the SCDD, the measurements were repeated after 0.25–4 h intervals to investigate the ‘destabilization’.

## RESULTS

**Fig. 2.**
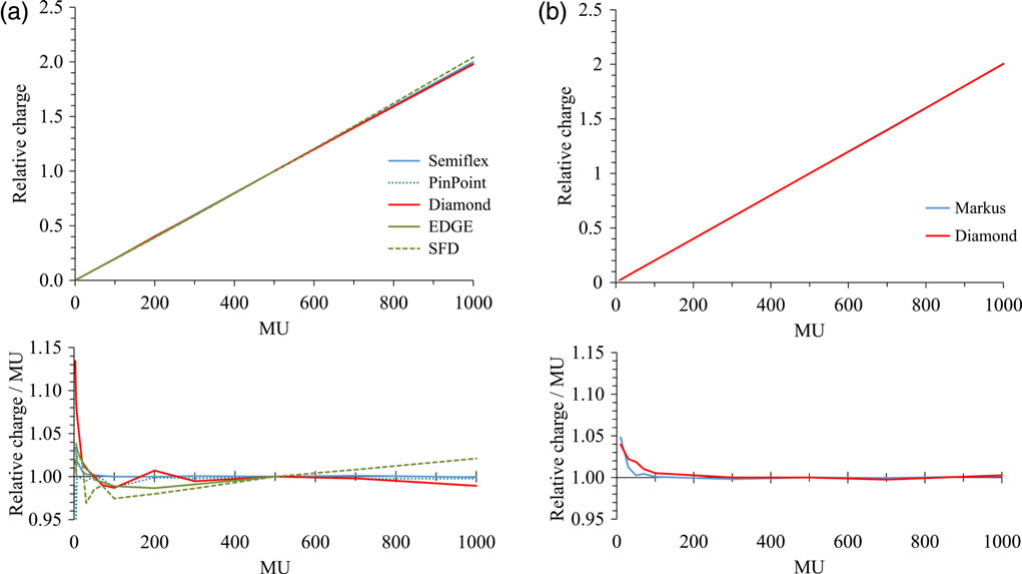
Dose linearity of (**a**) a 6-MV photon beam and (**b**) a uniform-scanning proton beam. For each detector, the dose/MU is plotted in the lower graphs. Values are normalized at 500 MU.

Figure 2a shows the dose linearity evaluated for the 6-MV photon beam measured with a 500 MU/min dose rate. The linearity of the SCDD was within 1% except for at small MU. The values of SFD became larger than those of other detectors with large MU. Figure 2b illustrates the dose linearity evaluated for the proton beam. SCDD showed excellent linearity for ≥100 MU. Although the values increased at <100 MU, the values of the Markus chamber also increased, representing the stability issue of the beam output but not the dose dependency of the detectors.

**Fig. 3.**
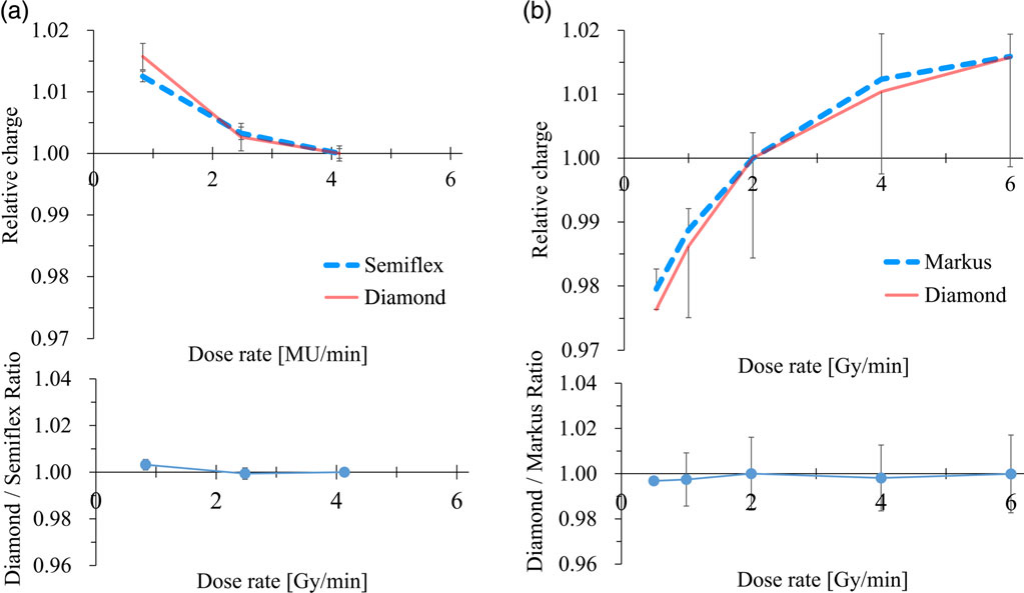
The relative charge and the ratio between the data of the diamond and the ion chamber are plotted against dose rate and shown in the upper and lower graphs, respectively. (**a**) A 6-MV photon beam. Values are normalized at that of a 500 MU/min dose rate. (**b**) Uniform-scanning proton beam with an energy range of 16 cm and 10 cm SOBP. Values are normalized at that of 2 Gy/min dose rate.

Figure 3a shows the dose-rate dependency against 100 MU of 6-MV photon beam. Both the Semiflex ion chamber and the SCDD showed an increase in charge of up to 1.6% with decreasing dose rate, representing the machine characteristics but not the dose-rate dependency of each chamber. The difference between these two chambers was <0.3%. Figure 3b shows the relative response of the SCDD and the Markus chamber against proton beams with various beam currents. Although dose-rate-dependent variation was observed, the differences between the Markus and diamond detectors were within 1% in the range of 0.5–6 Gy/min dose rate, representing the variation in beam output but not the dose-rate dependency of the detectors.

**Fig. 4.**
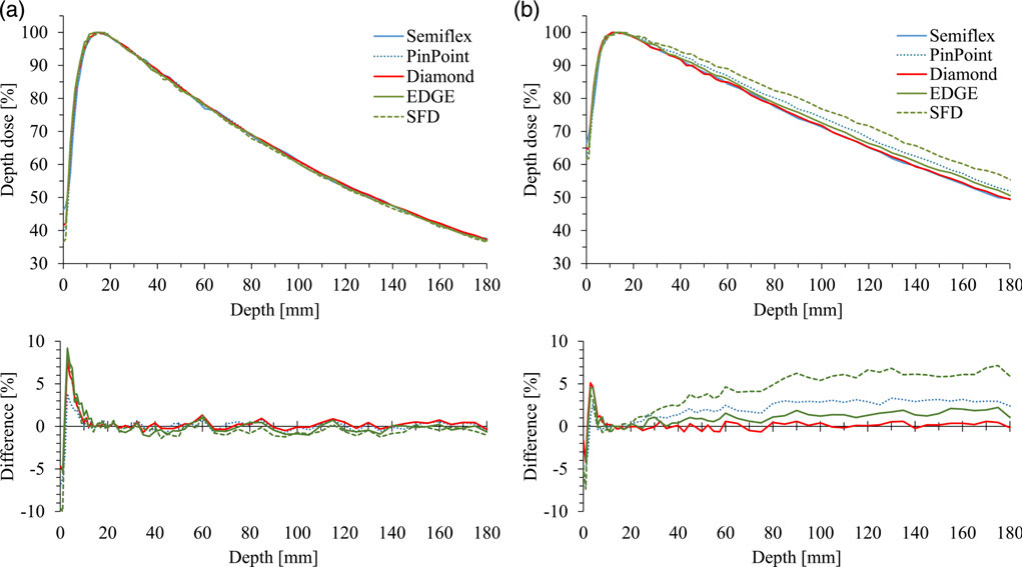
Percentage depth–dose of a 6-MV photon beam of (**a**) 3 × 3 cm^2^ and (**b**) 30 × 30 cm^2^ field sizes. For each data, the difference from Semiflex chamber is plotted in the lower graphs. Note that the diamond data has the smallest difference at depths and field size.

Figure 4a and b show the PDD of the 6-MV photon beam with 3 × 3 cm^2^ and 30 × 30 cm^2^ field sizes, respectively. Percentage differences relative to the value of the Semiflex ionization chamber were also plotted in the lower graphs. For 3 × 3 cm^2^ field size, all detectors showed similar results, with the differences within 1.5% at the region deeper than *d_max_*. For 30 × 30 cm^2^ field size, in contrast, the values of SFD showed a difference of up to 7.1%. The PinPoint chamber and EDGE detector also showed differences of up to 2.2% and 3.3%, respectively. The SCDD showed a small difference (within 0.6%). Similar variability among detectors are known and reported in TG-106 [31].

**Fig. 5.**
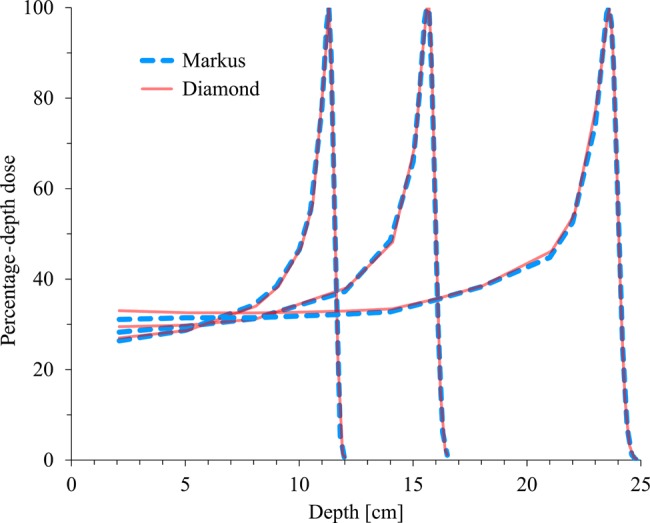
Percentage depth–dose of proton beams with the energy range of 8, 16 and 24 cm.

Figure 5 illustrates the PDD of the pristine Bragg peak of the proton beam with the energy range of 8, 16 and 24 cm. The data measured with the SCDD showed very similar data, representing the small energy-dependency of the SCDD against the proton beams.

**Fig. 6.**
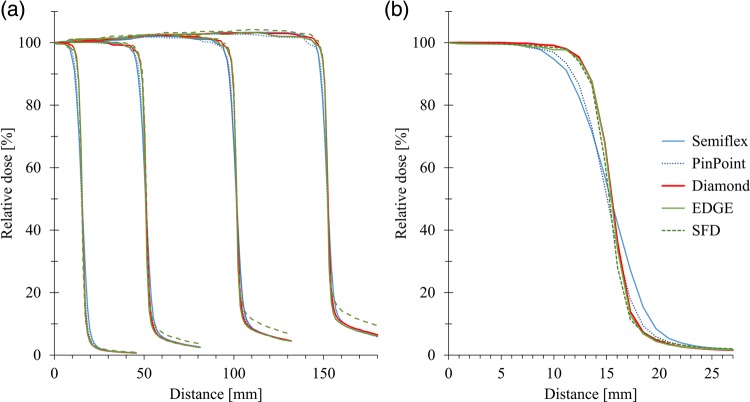
(**a**) Profiles of 6-MV photon beams of 3 × 3 cm^2^, 10 × 10 cm^2^, 20 × 20 cm^2^, and 30 × 30 cm^2^ field size measured at d_max_. (**b**) Profiles of 3 × 3 cm^2^ field size are focused. Note the diamond detector provides a superior profile.

Figure 6a shows the OCR of the 6-MV X-ray beam with various field sizes measured at *d_max_*. For large field sizes, only the SFD showed larger values in the ‘tail’ region. Figure 6b illustrates the OCR of 3 × 3 cm^2^ field size. The diamond, EDGE detector and SFD showed almost identical profiles with steep penumbrae. The Semiflex chamber showed an averaging effect at the penumbrae region. Although the PinPoint chamber showed a slightly better profile, the averaging effect is still observed.

**Fig. 7.**
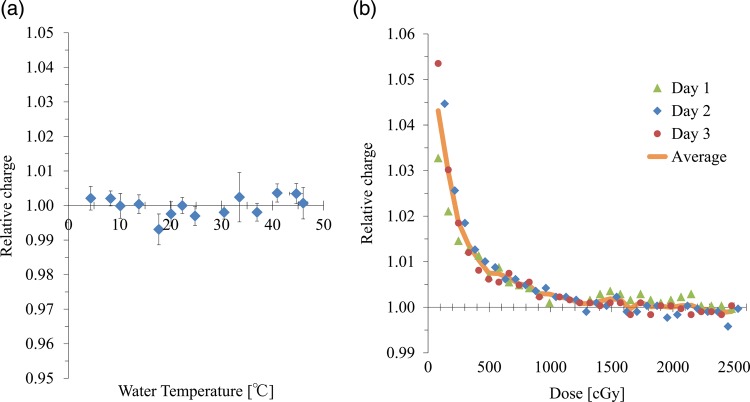
(**a**) Charge measured for 100 MU of 6-MV photon beams with various water temperatures. Values are normalized by the value at 22°C. Horizontal bars represent the range of the temperature recorded before and after measurements. (**b**) Effects of the pre-irradiation. The data are normalized by the average value in the range between 2000 and 3000 MU.

The temperature dependence is shown in Fig. 7a for the response of diamond detector against 100 MU of a 6-MV photon beam. Values were normalized at 22°C. In the range of 4–41°C, the differences were within 0.7%. At temperatures ≥44°C, a large signal was observed without irradiation. Although the values were corrected by subtracting the average value of the leakage for the beam-on time (12 s for 500 MU/min), the measured values at temperatures >50°C will not be correct.

Figure 7b shows the stabilization of the diamond chamber by pre-irradiation. Values were normalized by the average value in the range between 2000 and 3000 MU of three series of measurements. The response of the initial irradiation was, on average, 4.3% higher than the value at plateau. A dose of 900 cGy and 1200 cGy was needed to stabilize the chamber to the level within 0.5% and 0.2%, respectively. At 0.25–4 h after pre-irradiation, the measurements were repeated to assess the destabilization. The charges measured for the initial 100 MU of irradiation were 0.9%, 1.6% and 2.2% higher than the values at plateau for 0.25-, 1- and 4-h intervals, respectively.

## DISCUSSION

Several studies have investigated the characteristics of this commercial diamond detector, especially for electron-beam and small-field photon-beam dosimetry. For proton-beam dosimetry, Mandapaka *et al.* previously investigated the characteristics of the prototype SCDD with the PMMA housing [20]. However, the characteristics of the commercial model 60019 for proton-beam dosimetry have not been reported. In this study, we have investigated the characteristics of the commercial SCDD type 60019 for the dosimetry of a uniform-scanning proton beam. As shown in Fig. 2, the SCDD showed similar results for dose linearity to those of the parallel-plate ionization chamber. Although the response was slightly large at doses <100 MU, the result will be due to the beam output stability, since the ionization chamber showed similar results. As illustrated in Figs 3 and 5, the SCDD showed good characteristics for proton dosimetry in terms of the dose rate and energy dependency. Mandapaka *et al.* also reported small dose-rate dependency of the SCDD and good agreement for PDD measured with the ion chamber and the SCDD [20].

For photon-beam dosimetry, Chalkley *et al.*, Morales *et al.* and Laub *et al.* investigated the dosimetric characteristics of the commercial SCDD model 60019 for the CyberKnife, for the Novalis Trilogy linac and for the Elekta Synergy linac, respectively [26–28]. In this study, we investigated the dose linearity, dose-rate dependency, PDD, and profile of the Varian Clinac 2100C/D linac. As illustrated in Figs 2–4, the SCDD showed excellent characteristics for photon-beam dosimetry in terms of the consistency of response and dose-rate dependency, as supported by previous reports. As shown in Fig. 6, the SCDD showed a steep profile penumbra, similar to the EDGE and SFD diode detectors, indicating small volume-averaging effects. At the tail region, the SCDD showed a similar profile to the ionization chambers, indicating low energy dependency. The sensitive volume is circular shape, with 1.1 mm radius and 1 μm thickness. Ciancaglioni *et al.* showed that horizontal setting of SCDD to the beam axis showed better resolution of the beam profile measurements [19]. With horizontal set-up of the SCDD, the averaging effects can be minimized.

The commercial SCDD showed a stable response in the temperature range of 4–41°C, with variation within ±0.7% (Fig. 7a). Ciancaglioni *et al.* investigated the prototype SCDD embedded in the PMMA housing in the 18–40°C range and reported the temperature dependency within 0.2% [19]. They also reported that 60 cGy of pre-irradiation was necessary for the prototype SCDD in order to stabilize the detector response within ±0.5%. Di Venanzio *et al.* reported that the response of the prototype SCDD embedded in the waterproof housing of a PTW type 60017 diode detector was stabilized by 500 cGy of electron beam from 0.7% to 0.1% [24]. Laub *et al.* reported that the response of the microDiamond detector was within 0.5%, without any pre-irradiation [26]. They concluded that ∼300 cGy of pre-irradiation would be sufficient. In contrast, our study showed that a dose of 900 cGy and of 1200 cGy was needed to stabilize the chamber to the level within 0.5% and 0.2%, respectively (Fig. 7b). For the detector we used, at least 1000 cGy of pre-irradiation is recommended in order to conduct accurate measurements. In addition, pre-irradiation is recommended after a few hours, indicating the importance of the characterization of each detector before usage for data collection.

## CONCLUSION

We investigated the characteristics of the new commercial SCDD type 60019 for uniform-scanning proton-beam dosimetry. The SCDD showed sufficient constancy, similar to that of the ionization chamber. The SCDD also showed significantly better characteristics than other detectors for radiation dosimetry in terms of excellent spatial resolution and stability of response. However, at least 1000 cGy of pre-irradiation will be needed for accurate measurements.

## FUNDING

Funding to pay the Open Access publication charges for this article was provided by PTW-Freiburg, Germany. akino-radonc@umin.net.
